# LINC01354 interacting with hnRNP-D contributes to the proliferation and metastasis in colorectal cancer through activating Wnt/β-catenin signaling pathway

**DOI:** 10.1186/s13046-019-1150-y

**Published:** 2019-04-15

**Authors:** Jing Li, Meirong He, Wen Xu, Silin Huang

**Affiliations:** 1 Guangdong Provincial Key Laboratory of Gastroenterology, Department of Gastroenterology, Nanfang Hospital, Southern Medical University, Guangzhou, 510515 Guangdong Province China; 2 Department of Ultrasound, The Third Affiliated Hospital of Southern Medical University, Guangzhou, 510000 Guangdong Province China

**Keywords:** LINC01354, hnRNP-D, Wnt/β-catenin, mRNA stabilization, CRC

## Abstract

**Background:**

Long non-coding RNAs (lncRNAs) have been identified to play an important role in the development and progression of various tumors, including colorectal cancer (CRC). However, the regulatory molecular mechanism by lncRNA in CRC initiation and progression has not been fully clarified.

**Methods:**

TCGA database was used to identify the involvement of LINC01354 in CRC. qRT-PCR and western blot were used to determine RNA and protein expression. The gain- and loss-of-function assays were conducted to explore the function of LINC01354 in the progression of CRC. In order to investigate the LINC01354-mediated mRNA in CRC tumorigenesis, we applied the profiling analysis as well as GO and KEGG analysis. Pulldown and RIP assays were applied to detect the interaction of hnRNP-D with LINC01354 and β-catenin.

**Results:**

The upregulation of LINC01354 in CRC and its prognostic significance were identified by TCGA database and confirmed in CRC tissues. Functionally, forced expression of LINC01354 promoted, while knockdown of LINC01354 inhibited cell proliferation, migration and EMT phenotype formation of CRC cells. A significant enrichment of the Wnt/β-catenin signaling pathway genes under LINC01354 overexpression. In addition, LINC01354 modulated the mRNA stability of β-catenin through interacting with hnRNP-D, thereby activating Wnt/β-catenin signaling pathway.

**Conclusions:**

Our investigations proposed novel regulatory axis of LINC01354/hnRNP-D/Wnt/β-catenin, which might be in favor of exploring novel therapeutic regimens for the clinical treatment of CRC.

**Electronic supplementary material:**

The online version of this article (10.1186/s13046-019-1150-y) contains supplementary material, which is available to authorized users.

## Introduction

Colorectal cancer (CRC) is one of the most common malignancies recognized as the third leading cause of cancer-related deaths worldwide [1]. Despite great efforts dedicated in understanding the underlying pathomechanism and exploring novel therapeutic strategies, the prognosis of advanced-stage CRC patients remains far from satisfactory due to the ineffectiveness of diagnostic methods, early metastasis and easily recurrence. Tumor metastasis is an intricate process involving multiple genetic and epigenetic alterations, leading to the activation or inactivation of tumor suppressors or oncogenes [2]. Although multiple carcinogens and varying genetic backgrounds have been identified in the initiation and progression of CRC, the detailed intermolecular mechanisms and regulation of key pathways implicated in the progression of this disease are still obscure. Therefore, it is urgent to identify the molecular mechanism of CRC progression and metastasis.

With the development of high-throughput sequencing, the transcription of short or long noncoding RNAs (lncRNAs) from human genome has been uncovered, representing a great breakthrough [3, 4]. LncRNAs, with a length > 200 nt, have no or limited protein coding capacity [5, 6]. Based on its location to the nearby coding genes, lncRNAs can be classified into five types including sense, antisense, intergenic, bidirectional and intronic [7]. Emerging evidences have documented the diverse roles of lncRNAs as a molecular modulator in regulating multiple biological process, such as guides, scaffolds, tethers and decoys [8–10]. For example, lncRNA PTTG3P contributes to the proliferation and metastasis of HCC cells through modulating PTTG1 via activating PI3K/AKT signaling in hepatocellular carcinoma [11]; Li D. et al. reported that Ets-1 promoter-associated lncRNA could drive the progression of gastric cancer through regulating NONO/ERG/Ets-1 axis [12]; Wang Y. et al. demonstrated that lncRNA DANCR, severing as a competitive endogenous RNA, contributes to ROCK1-mediated proliferation and metastasis through decoying of miR-335-5p and miR-1972 in osteosarcoma [13]. Long intergenic noncoding RNAs (lincRNAs), a class of transcript units discretely intervening between the protein-coding loci, have been characterized to exert important functions in multiple cellular processes, including tumorigenesis. For instance, lincRNAs termed HOTAIR and XIST have been reported in many cancers, including breast cancer, gastric cancer and glioblastoma, participating in a variety of important cellular processes, such as X chromosome inactivation, genomic imprinting, and so on [14–18]. Also, recent years witnessed the identification of a number of lincRNAs as key regulators in the initiation and progression of CRC [19, 20]. However, there remains a long way to the full understanding of the lincRNA-mediated regulatory mechanism behind in CRC pathogenesis and progression.

In the present study, we confirmed the oncogenic role of LINC01354 in CRC and its prognostic potential in CRC patients. And it was observed that LINC01354 knockdown weakened the growth and metastasis ability of CRC cells. We further validated that LINC01354 could interact with heterogeneous ribonucleoprotein D (hnRNP-D) protein, which therefore contributed to the activation of Wnt/β-catenin signaling pathways in CRC cells. Our investigations provide novel insights into the biological function and molecular regulatory mechanisms of LINC01354 in CRC pathogenesis and identify LINC01354 as a novel potential prognostic biomarker and therapeutic target for CRC intervention.

## Materials and methods

### Clinical specimens

CRC specimens and adjacent normal tissues (*n* = 88) were obtained from 88 patients who received surgery in Nanfang Hospital. None of the patients received any treatment before surgery. All the samples were immediately frozen in liquid nitrogen and stored at − 80 °C until use. Each patient was returned for follow-up visit every 3 months. The clinical and pathological characteristics were obtained from patients’ history record. Overall survival time was defined from the date of operation to the date of death or last contact.

### Cell culture

Human CRC cell lines (HCT116, HT29, SW620, SW480 and LoVo) and normal colorectal epithelium cell line FHC were obtained from the American Type Culture Collection (Manassas, VA, USA). The SW480 cells were cultured in RPMI 1640 medium supplemented with 10% FBS (Gibco) while others in McCoy’s 5A complete medium (Gibco). All the cell lines were routinely authenticated with the interval of 6 months by cell morphology monitoring and growth curve analysis. All cell lines were cultured in a humidified chamber with 5% CO_2_ at 37 °C.

### Cell transfection

To knockdown or overexpress LINC01354 in LoVo or HCT116 cells, shRNA (siLINC01354#1 and siLINC01354#2) or pcDNA3.1/LINC01354 were used, with empty vectors as negative control. All vectors were obtained from Shanghai Integrated Biotech Solutions Co., Ltd. (Shanghai, China) and transfected into cells utilizing Lipofectamine 2000 (Invitrogen, Carlsbad, CA, USA) under manufacturer’s protocols. The efficiency of transfection was evaluated by qRT-PCR.

### Cell proliferation assays

For CCK-8 assay, CRC cells were seeded into 96-well plates at a density of 1 × 10^3^ per well. Cell Counting Kit-8 (CCK-8; Dojindo Laboratories, Kumamoto, Japan) was added into each well at indicated time points. After incubation at 37 °C for 2 h, the absorbance was measured using an automatic microplate at 450 nm.

For EdU (5-ethynyl-2′-deoxyuridine) assay, exponentially growing cells plated on 6-well plates (Corning) were labelled as described [21]. Then EdU-labeled or unlabeled LoVo and HCT116 cells were cultured into a 96-well plate at 2 × 10^3^ cells/well in McCoy’s 5A medium added with 10% FBS at 37 °C. At 0, 24, 48, 72, and 96 h, 10 μl of Cell Titer-96 reagent (Promega Inc., Madison, WI) was supplemented to each well. After 1 h of further incubation at 37 °C, the cells were scanned at the wavelength of 490 nm in a plate reader (Molecular Devices Corp., Sunnyvale, CA).

### Apoptosis assays

Cells (1 × 10^5^/well) were collected 48 h after transfection and were stained with Annexin V-FITC and propidium iodide (PI) according to the manufacturer’s instructions (BD Biosciences, Erembodegem, Belgium). Flow cytometry (BD FACSCalibur; BD Biosciences) was utilized to detect the apoptosis rate of transfected LoVo and HCT 116 cells by determining the relative amount of Annexin V-FITC-positive-PI-negative cells.

### Transwell assays

For invasion assay, cells in non-serum McCoy’s 5A complete medium (Gibco) were placed into the top chambers coated with 100 μl Matrigel, and McCoy’s 5A complete medium (Gibco) containing 20% FBS was supplemented to the bottom chamber. Post 24 h, Matrigel glue and extra cells in the upper chamber were removed by cotton swabs. Methanol was applied to confirm the membranes for 10 min. The cells invading to the lower membrane were calculated following crystal violet staining. Procedures of migration assay was as described above except for the Matrigel free upper insert.

### RNA pull down assay

Biotin-labeled RNAs were obtained by the MaxiScript T7 kit (Ambion). Biotin-labeled RNAs in refolding buffer (10 mM Tris pH 7.5, 0.1 M KCl and10 mM MgCl_2_) were added into cell lysates and incubated overnight at 4 °C with rotation, followed by addition of washed streptavidin-Dyna beads (Dyna beads M-280 Streptavidin, #11205D, Invitrogen). After being washed 4 times, beads were boiled and precipitated proteins were checked with western blot.

### RNA immunoprecipitation (RIP) assay

Cells were isolated and lysed with RIP buffer (20 mM Tris pH 7.5, 150 mM NaCl, 1 mM MgCl_2_, 0.1% NP40, 5% glycerol and 0.5 mM DTT) supplemented with RNase inhibitor, followed by addition of mouse anti-hnRNP-D (Abcam) with rabbit anti-IgG (Abcam) as control. RNA-protein complexes were enriched by the magnetic beads conjugated with anti-hnRNP-D. Then precipitated RNAs were eluted and used for cDNA synthesis by qRT-PCR.

### Luciferase reporter gene assays

CTNNB1 wild type with potential LINC01354 binding sites or relative mutant sites was generated by Sangon Biotech (Shanghai, China) and fused with the luciferase reporter vector psi-CHECK-2 (Promega, Madison, WI, USA). Cells were co-transfected with luciferase plasmids and siLINC01354 or siNC using the Lipofectamine 2000 under the manufacturer’s protocols. After 48 h of transfection, dual luciferase assay kit (Promega, Madison, WI, USA) was applied to measure the luciferase activity. Renilla luciferase activity acted as a normalization control.

### Fluorescence in situ hybridization (FISH)

RNA FISH was performed on the HCT116 or LoVo cells arrays following the protocol described in Raj et al. Nat Meth 2008 [22] with minor adjustment. Briefly, cells were pre-washed with the washing buffer containing 10% formamide and 2× saline-sodium citrate (SSC). Then the hybridization was performed by adding 1 μL of probe into 50 μL of hybridization buffer with 10% formamide, 2× SSC, and 10% dextran sulfate (*w*/*v*). The final concentration of probe was 3.5 μM for LINC01354 probe for the overnight hybridizations in a humidified chamber at 37 °C. After hybridization, the samples were washed twice and then imaged in 2× SSC.

### TOP-flash luciferase assay

LoVo cells were planted in 24-well culture plates and serum-starved overnight. The siLINC01354 or siNC were then co-transfected with TOP flash plasmid or FOP flash plasmid into cells using Lipofectamine 2000. Post 48 h of transfection, the Dual Luciferase Assay Kit was applied to determine the activities of both firefly and Renilla luciferase reporters. The TOP-Flash reporter activity was presented as the relative proportion of luciferase activity to Renilla activity.

### Statistics

SPSS version 20.0 software (SPSS Inc., Chicago, IL, USA) was used for statistical analysis. Data was expressed as mean ± SD. The Kaplan-Meier analysis and log-rank test were used to calculate the overall survival. One-way ANOVA analysis or two-tailed Student’s t-tests were performed for *p*-value analysis, as appropriate. *p* < 0.05 was considered statistically significant. Every experiment was repeated in triplicate.

## Results

### LINC01354 was identified from online database analysis

**Fig. 1:**
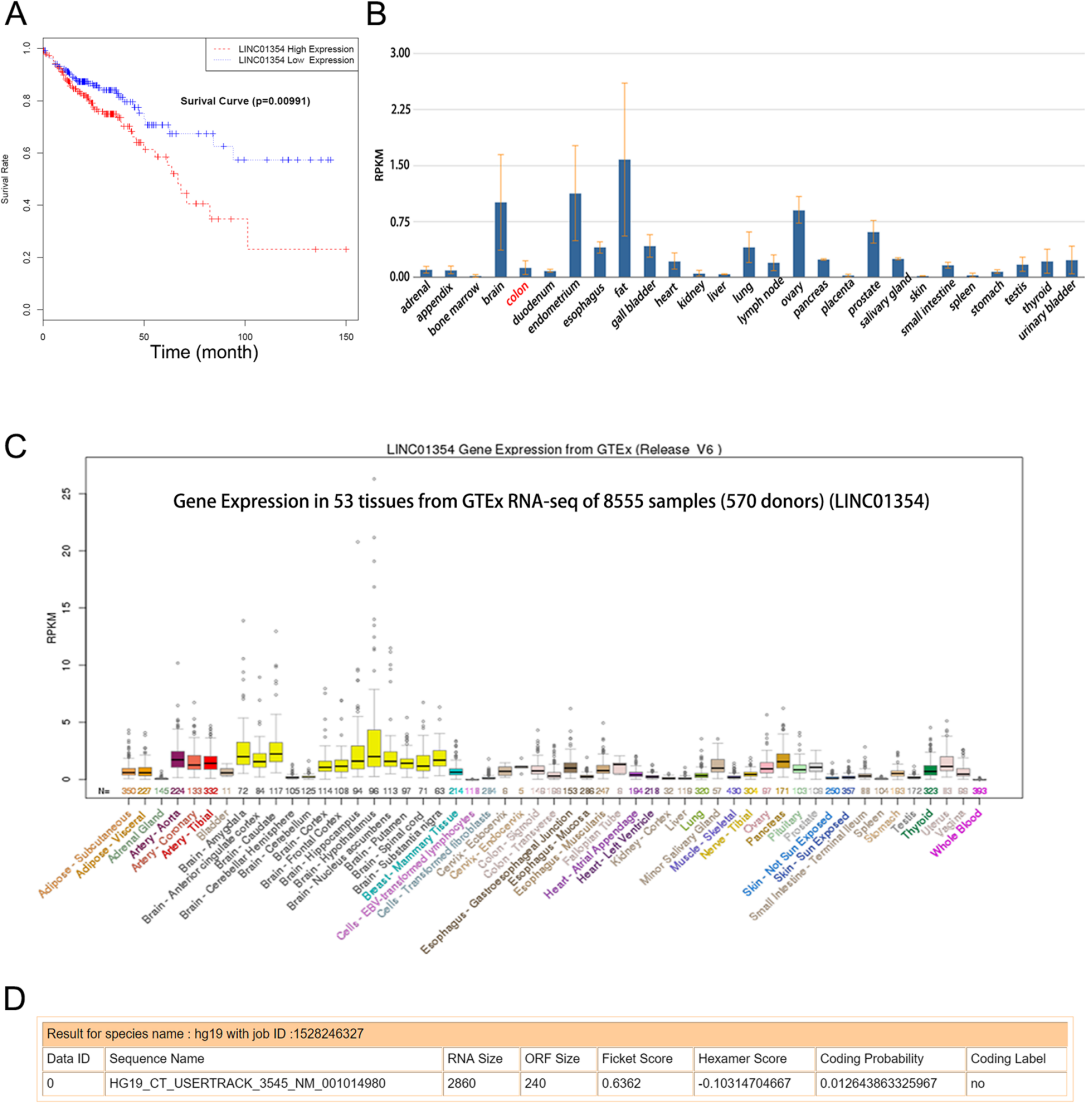
LINC01354 was identified from online database analysis. **a** The Cancer Genome Atlas (TCGA) data analysis of the correlation between expression of LINC01354 and overall survival rate. **b**-**c** NCBI and UCSC results of down-expression of LINC01354 in normal colon tissues. **d** Coding ability of LINC01354 was examined by online coding potential assessment tool (http://lilab.research.bcm.edu/cpat/index.php)

To probe the lncRNA-mediated initiation and progression of CRC, we analyzed several online databases. Based on the analysis of The Cancer Genome Atlas (TCGA) data, we found that high expression of LINC01354 predicted poor outcomes in CRC cases (Fig. 1a). What’s more, based on the results from NCBI (https://www.ncbi.nlm.nih.gov/), significant downregulation of LINC01354 was identified in normal colon tissues (Fig. 1b), which was consistent with the results by analyzing data from UCSC (http://www.ucsc.edu/) online database (Fig. 1c). Finally, to assess whether LINC01354 was a bona fide lncRNA, we applied online coding potential assessment tool (http://lilab.research.bcm.edu/cpat/index.php) and found that the full size of LINC01354 was 2860 nt and the ORF size was 240, and that the coding probability of LINC01354 was 0.01264 (Fig. 1d). All these online data revealed that LINC01354, as a long non-coding RNA, potentially predicted poor outcomes of CRC cases and was low expressed in normal colon tissues, indicating its potential oncogenic role in CRC.

### LINC01354 was upregulated in the clinical CRC specimens and cell lines

**Fig. 2:**
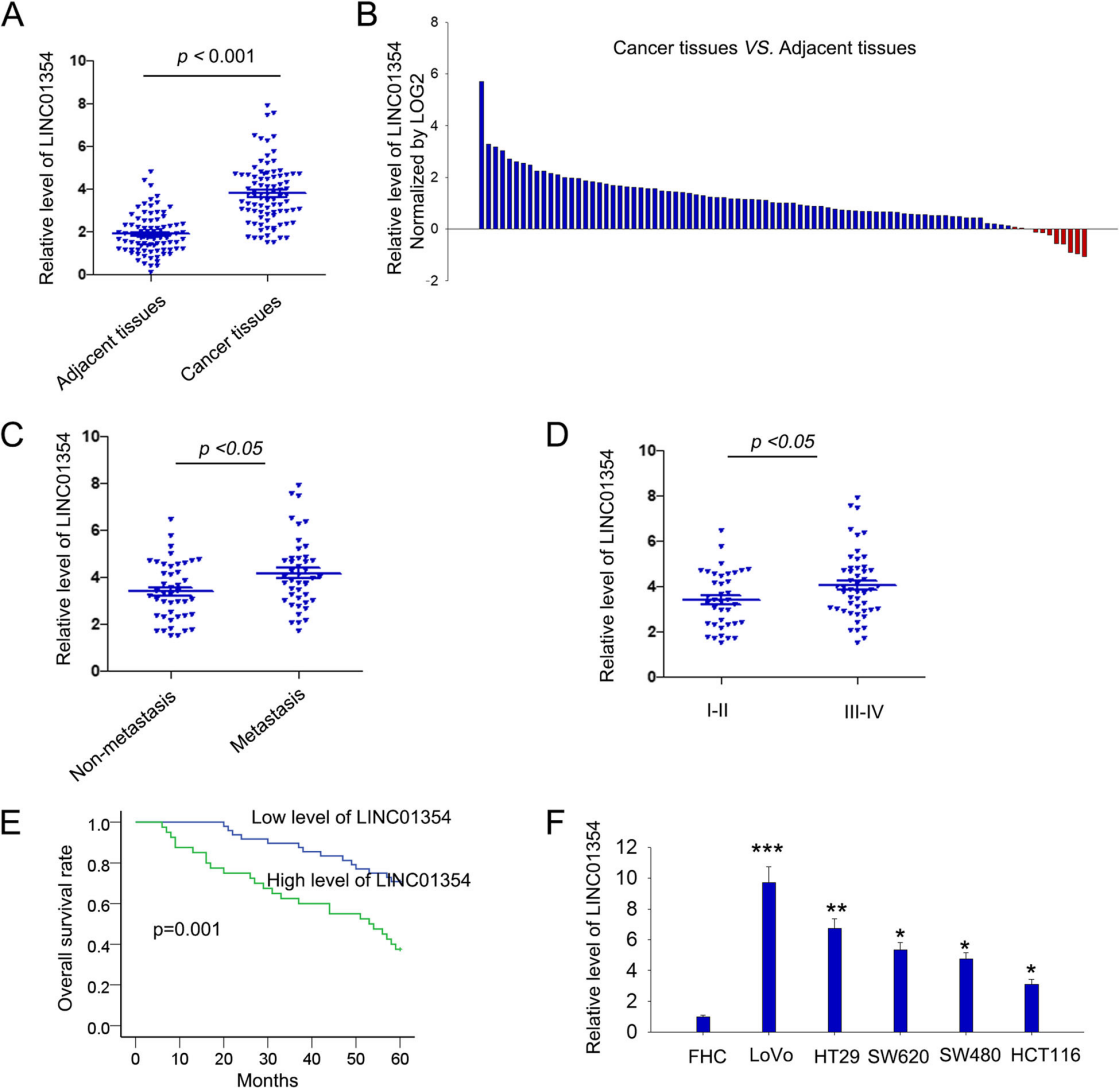
The upregulation of LINC01354 in the clinical CRC samples and cell lines. **a** qRT-PCR analysis of the expression levels of LINC01354 in 88 paired CRC tissues and the corresponding adjacent normal tissues. **b** LINC01354 expression level was analysed in 88 CRC samples and expressed as log_2_ fold change (cancer/normal). **c** The expression levels of LINC01354 in tissues with or without metastasis were accessed by qRT-PCR. **d** LINC01354 expression in different TNM stages of CRC was evaluated by qRT-PCR. **e** Kaplan-Meier analysis and log-rank test were employed to estimate the relation between LINC01354 expression and overall survival of CRC patients. **f** qRT-PCR results of LINC01354 expression in normal colorectal epithelium cell line FHC and five CRC cell lines (LoVo, HT29, SW620, SW480, and HCT116), of which LoVo cells exhibited the highest LINC01354 expression and HCT116 cells showed the lowest LINC01354 levels. All of the cell lines were analyzed from data of three independent experiments. ^*^p < 0.05, ^**^p < 0.01, and ^***^p < 0.001 vs. control group

To further investigated the role of LINC01354 in CRC tumorigenesis, we collected clinical CRC specimens to measure the expression levels of LINC01354 in 88 paired CRC tissues and the corresponding adjacent normal tissues by qRT-PCR. It was discovered that LINC01354 expression was higher in 45 of 88 (51.13%, Fold change > = 2) CRC specimens in comparison to the adjacent normal mucosa tissues (*p* < 0.001) (Fig. 2a-b). Furthermore, to probe the clinicopathologic significance of LINC01354, LINC01354 expression was divided into a high-expression group (*n* = 44) and a low-expression group (*n* = 44) based on the cutoff value (the median level of LINC01354, cutoff value = 2.412). As shown in Table 1, LINC01354 expression was positively associated with tumor size, lymph metastasis, tumor-node-metastasis (TNM) stage, and distant metastasis in CRC. Additionally, metastatic tissues exhibited higher LINC01354 expression than non-metastatic tissues (Fig. 2c, *p* < 0.05). And through multivariate analysis of prognostic parameters in CRC patients, it was found that only distance metastasis and LINC01354 expression could serve as independent markers for CRC prognosis **(**Table 2**)**. The patients at TNM I-II showed lower LINC01354 expression than patients at TNM III-IV (Fig. 2d, *p* < 0.05). Kaplan-Meier survival analysis of CRC patients showed that higher LINC01354 expression in CRC patients suggested poorer overall survival (OS) (Fig. 2e, *p* = 0.001). Moreover, we evaluated the levels of LINC01354 in one normal colorectal epithelium cell FHC and five CRC cell lines (LoVo, HT29, SW620, SW480, and HCT116), finding that compared with FHC normal colorectal epithelium cells, the expression levels of LINC01354 were up-regulated in LoVo, RKO, HT29, SW620, SW480, and HCT116 cell lines, among which LoVo cells exhibited highest LINC01354 expression, whereas HCT116 the lowest (Fig. 2f). All these findings revealed that LINC01354 may act as an oncogene in CRC.

**Table 1:** Correlation between LINC01354 Expression and Clinical Features. (*n* = 88)

Variable	Groups	LINC01354 expression		*P*-Value
		Low	High	
Age	<60	28	26	0.6605
	> = 60	20	14	
Gender	Male	16	11	0.6450
	Female	32	29	
Tumor Location	Rectum	22	17	0.8307
	Colon	26	23	
Tumor Size	<3	29	11	0.0026^**^
	> = 3	19	29	
Lymph Node Metastasis	No	24	10	0.0272^*^
	Yes	24	30	
TNM	I, II	26	12	0.0308^*^
	III, IV	22	28	
Distance	No	30	14	0.0180^*^
Metastasis	Yes	18	26	

Low/high by the sample median. Pearson χ^2^ test. *P* < 0.05 was considered statistically significant. ^*^ *P* < 0.05; ^**^P < 0.001

**Table 2:** Multivariate analysis of prognostic parameters in patients with colorectal cancer by Cox regression analysis

Variable	Group	HR	CI (95%)	*P*-Value
Age	<60	0.607	0.298–1.236	0.169
	> = 60			
Gender	Mail	1.295	0.577–2.908	0.531
	Female			
TumorLocation	Rectum	0.833	0.43–1.615	0.589
	Colon			
TumorSize	<3	1.572	0.774–3.193	0.211
	> = 3			
Lymph Node	No	2.823	1.294–6.162	0.009^*^
Metastasis	Yes			
TNM	I,II	0.887	0.445–1.77	0.734
	III,IV			
DistanceMetastasis	No	2.15	1.089–4.246	0.027^*^
	Yes			
LINC01354	Low	2.264	1.106–4.634	0.025^*^
Expression	High			

Proportional hazards method analysis showed a positive, independent prognostic importance of Lymph Node Metastasis (*P* = 0.012) and LINC01354 expression (*P* = 0.023). *P < 0.05 was considered statistically significant

### LINC01354 promoted proliferation, migration, invasion and EMT of CRC in vitro

**Fig. 3:**
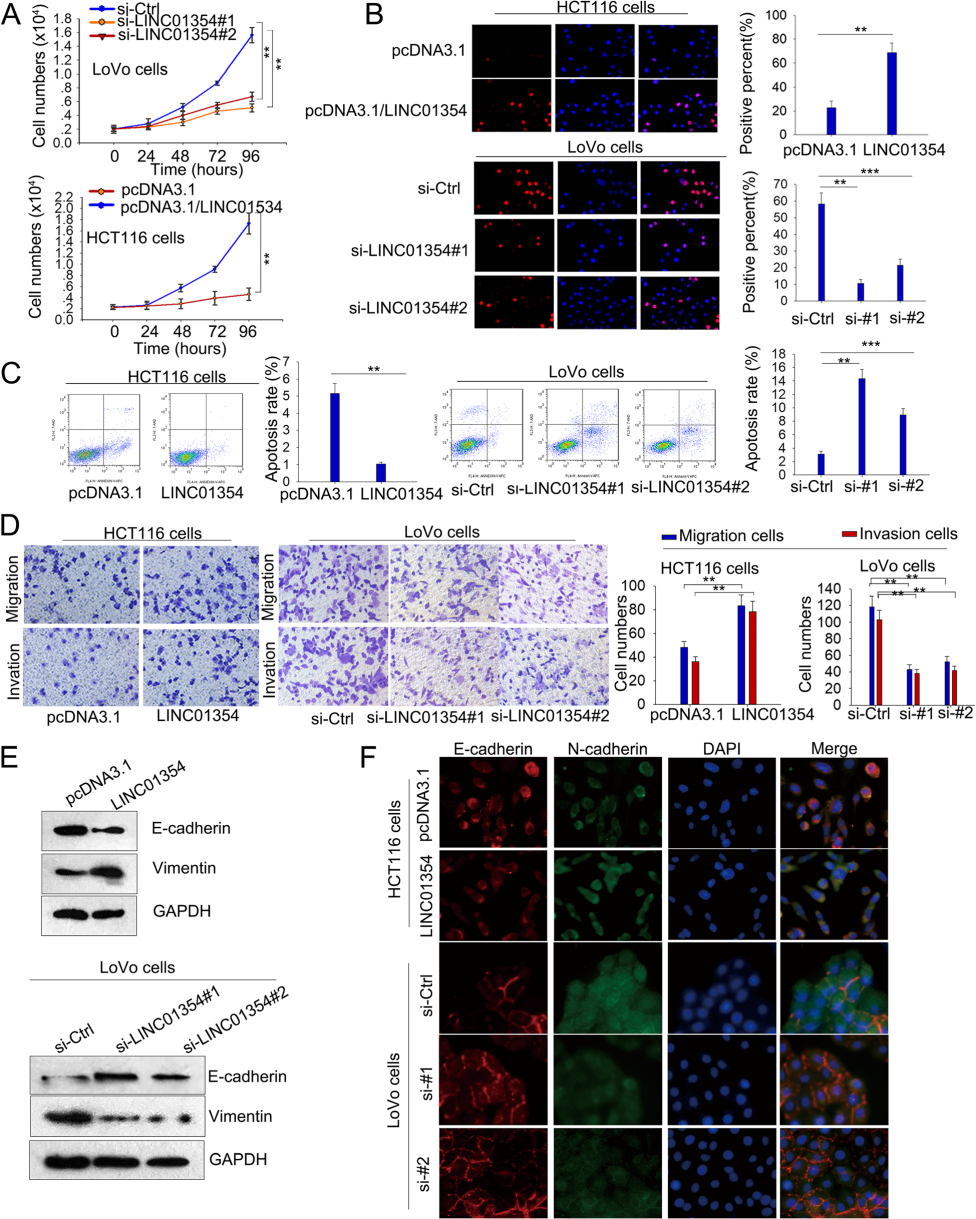
LINC01354 promoted proliferation and migration of CRC in vitro. **a**-**b** CCK8 and EdU assays were applied to examine cell proliferation ability in si-LINC01354#1/2 transfected LoVo cells and pcDNA3.1/LINC01354 transfected HCT116 cells. **c** The apoptosis rate in si-LINC01354#1/2 transfected LoVo cells and pcDNA3.1/LINC01354 transfected HCT116 cells was assessed by flow cytometry. **d** Transwell assays revealed the cell motility in LoVo cells transfected with si-LINC01354#1/2 and HCT116 cells transfected with pcDNA3.1/LINC01354. **e**-**f** Western blot and immunofluorescence were utilized to detect EMT associated proteins expression of E-cadherin and Vimentin in the two kinds of CRC cells. Results were exhibited as the mean ± SD on the basis of 3 independent experiments, ^**^p < 0.01, ^***^p < 0.001

To assess the biological effect of LINC01354 in CRC, we silenced LINC01354 with siRNA (siLINC01354#1 and siLINC01354#2) in LoVo cells and overexpressed LINC01354 in HCT116 cells with pcDNA3.1/LINC01354. Controls were infected with empty vectors. qRT-PCR assays were performed to determine the transfection efficiency (Additional file 1: Figure S1A). CCK8 proliferation assays elucidated that LINC01354 knockdown significantly weakened growth ability in LoVo cells, while LINC01354 overexpression enhanced growth ability in HCT116 cells (Fig. 3a). Consistently, results from EdU assays revealed that ratio of EdU positive cells was obviously reduced after the downregulation of LINC01354 in LoVo cells and was increased under the forced expression of LINC01354 in HCT116 cells (Fig. 3b). Furthermore, through assessing the apoptosis rate by flow cytometry, we found that siLINC01354#1 or siLINC01354#2-transfected cells presented higher apoptosis rate compared with the siCtrl transfected cells, while pcDNA3.1/LINC01354-transfected HCT116 cells represented opposite results (Fig. 3c). Additionally, the cell motility was measured by transwell assays, and the results revealed that the migratory and invasive ability was attenuated in LoVo cells transfected with either siLINC01354#1 or siLINC01354#2, whereas enhanced in HCT116 cells transfected with pcDNA3.1/LINC01354 (Fig. 3d). Moreover, knockdown of LINC01354 altered the epithelial-mesenchymal transition (EMT) related protein expression with E-cadherin increased and Vimentin decreased in siLINC01354#1/2-transfected LoVo cells, while opposite results were presented in HCT116 cells transfected with pcDNA3.1/LINC01354 (Fig. 3e-f). These findings revealed an inductive role of LINC01354 in proliferation, migration, invasion and EMT in CRC cells.

### Knockdown of LINC01354 inhibits tumor proliferation and metastasis in vivo

**Fig. 4:**
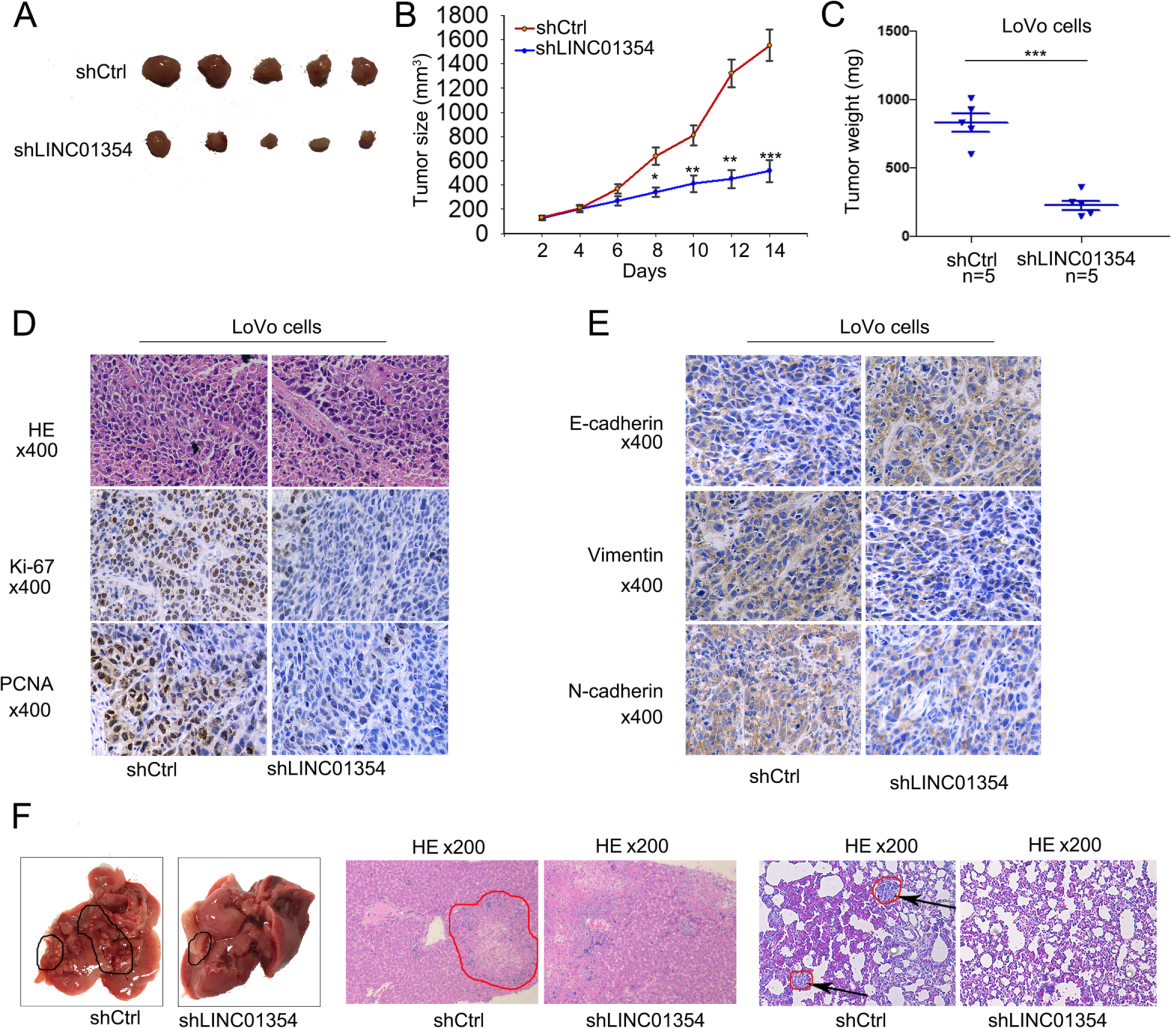
Knockdown of LINC01354 inhibits tumor proliferation and metastasis in vivo. **a** Images of tumors formed from mice injected with shCtrl or shLINC01354 transfected LoVo cells. **b**-**c** The shLINC01354 transfection decreased the tumor size and weight in vivo. **d** IHC assays revealed that the Ki-67 and PCNA proliferation index decreased in tumors originated from mice injected with LINC01354 silenced LoVo cells in comparison to tumors from mice injected with shCtrl transfected LoVo cells. **e** The expressions of E-cadherin and N-cadherin were determined by IHC experiments. **f** The metastatic nodes in lung in tumors originated from mice injected with LINC01354 silenced LoVo cells and tumors from that with shCtrl transfected LoVo cells. Data were showed as the mean ± SD. All assays were carried out in triplicate, ^*^p < 0.05, ^**^p < 0.01 and ^***^p < 0.001

To confirm the effect of LINC01354 on tumor proliferation in vivo, LoVo/shCtrl and LoVo/shLINC01354 cells were subcutaneously inoculated into the hind limb of nude mice. Tumor size was measured over time. LoVo/shLINC01354-injected mice developed smaller tumors than the LoVo/shCtrl-injected ones (*p* < 0.01; Fig. 4a-c). IHC assays confirmed that the Ki-67 and PCNA proliferation index in the LoVo/shLINC01354-xenografted tumors was weaker than that in LoVo/shCtrl-xenografted tumors (Fig. 4d). Moreover, tumors of LoVo/shLINC01354 group presented higher positive rate of E-cadherin, while lower positive rate of Vimentin compared with tumors of LoVo/shCtrl group (Fig. 4e). To determine the effect of LINC01354 on CRC metastasis in vivo, LoVo/shCtrl and LoVo/shLINC01354 cells were injected into the tail vein of mice. Eight weeks later, mice were killed, and lung and liver metastases were examined. Less metastatic nodules were obtained in the LoVo/shCtrl-xenografted tumors. However, LoVo/LINC01354-xenografted tumors had lung metastatic nodules (Fig. 4f). Consistent with the in vitro findings, these results suggested that LINC01354 significantly promoted tumor growth, metastasis and epithelial-mesenchymal transition (EMT) in vivo.

### Wnt/β-catenin pathway was involved in the function of LINC01354 in CRC cells

**Fig. 5:**
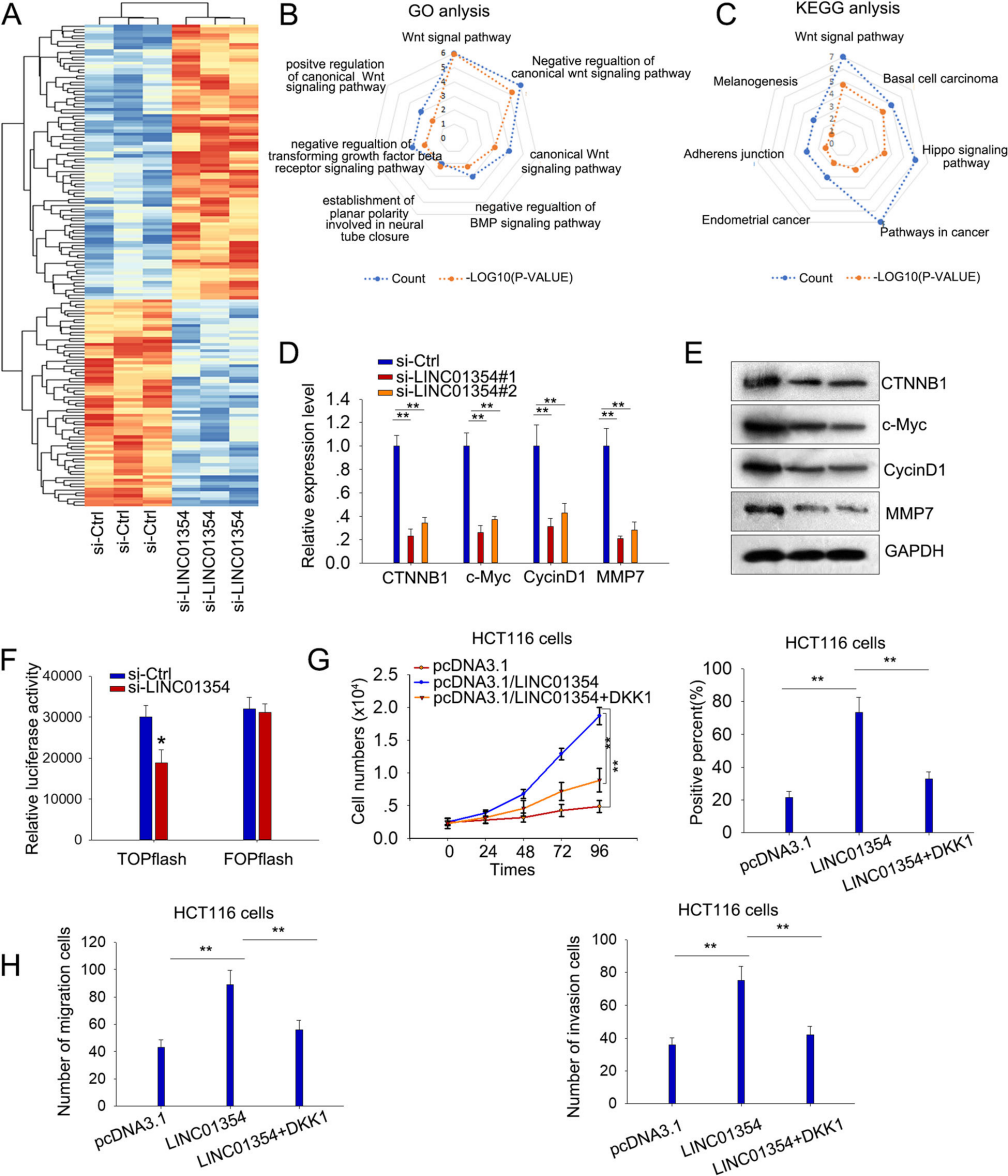
Wnt/β-catenin pathway was involved in the function of LINC01354 in CRC cells. **a** 67 downregulated genes under LINC01354 silencing were found by mRNA profiling analysis. **b**-**c** It was found by GO and KEGG analysis that the Wnt/β-catenin signaling pathway involved in the downstream pathway of LINC01354. **d**-**e** qRT-PCR and western blot analysis of the expression of genes (CTNNB1, c-Myc, CycinD1, and MMP7) involved in Wnt/β-catenin signaling pathway in LoVo cells with silencing of LINC01354. **f** The inhibitive effect of LINC01354 downregulation on Wnt/β-catenin pathway was detected by TOP-Flash luciferase reporter assay. **g** CCK8 and EdU assays were conducted to measure the impact of DKK1 on the pro-proliferation capacity of HCT116 cells transfected with pcDNA3.1/LINC01354. **h** The promoting function of pcDNA3.1/LINC01354 on migration and invasion of HCT116 cells was abrogated when exposed to DKK1 as screened by transwell assays. Results were shown as the mean ± SD on the basis of 3 independent experiments, ^*^p < 0.05, ^**^p < 0.01

In subsequence, we attempted to explore the mechanism through which LINC01354 regulated CRC. It has been known that lncRNAs participate in cancers though modulating gene expression and pathways [8–10], so we speculated that LINC01354 could regulate CRC through this way as well. Firstly, to identify the target genes modulated by LINC01354, we performed an mRNA profiling analysis in LINC01354-silenced LoVo cell lines. 67 genes were found statistically down-regulated more than two-fold in response to the knockdown of LINC01354 (Fig. 5a). We then conducted KEGG and GO analysis and identified the high enrichment score of Wnt/β-catenin signaling pathway among the downstream pathways of LINC01354 (*p* < 0.001; Fig. 5b-c). By applying qRT-PCR and western blot analysis, we confirmed the down-regulated mRNA and protein level of genes (CTNNB1, c-Myc, CycinD1, and MMP7) involved in Wnt/β-catenin signaling pathway in LINC01354-silenced LoVo cells (Fig. 5d-e). TOP-Flash luciferase assay revealed that LINC01354 downregulation inhibited Wnt/β-catenin pathway activity, certifying that LINC01354 played critical role in activating Wnt/β-catenin signaling (Fig. 5f). To further investigate whether LINC01354 regulated proliferation and metastasis in CRC cells through Wnt/β-Catenin signaling, pcDNA3.1/LINC01354-transfected HCT116 cells were treated with DKK1, the secreted antagonist of Wnt/β-Catenin signaling, for rescue assays. CCK-8 and EdU assays revealed that the pro-proliferation function of LINC01354 was partially vanished when treated with DKK1 (Fig. 5g). Besides, DKK1 treatment also abrogated the promoting function of LINC01354 overexpression on migration and invasion of HCT116 cells (Fig. 5h). These findings revealed that LINC01354 might exert oncogenic function at least partially through regulating Wnt/β-catenin pathway.

### LINC01354 positively regulated β-catenin through modulating the CTNNB1 stability

**Fig. 6:**
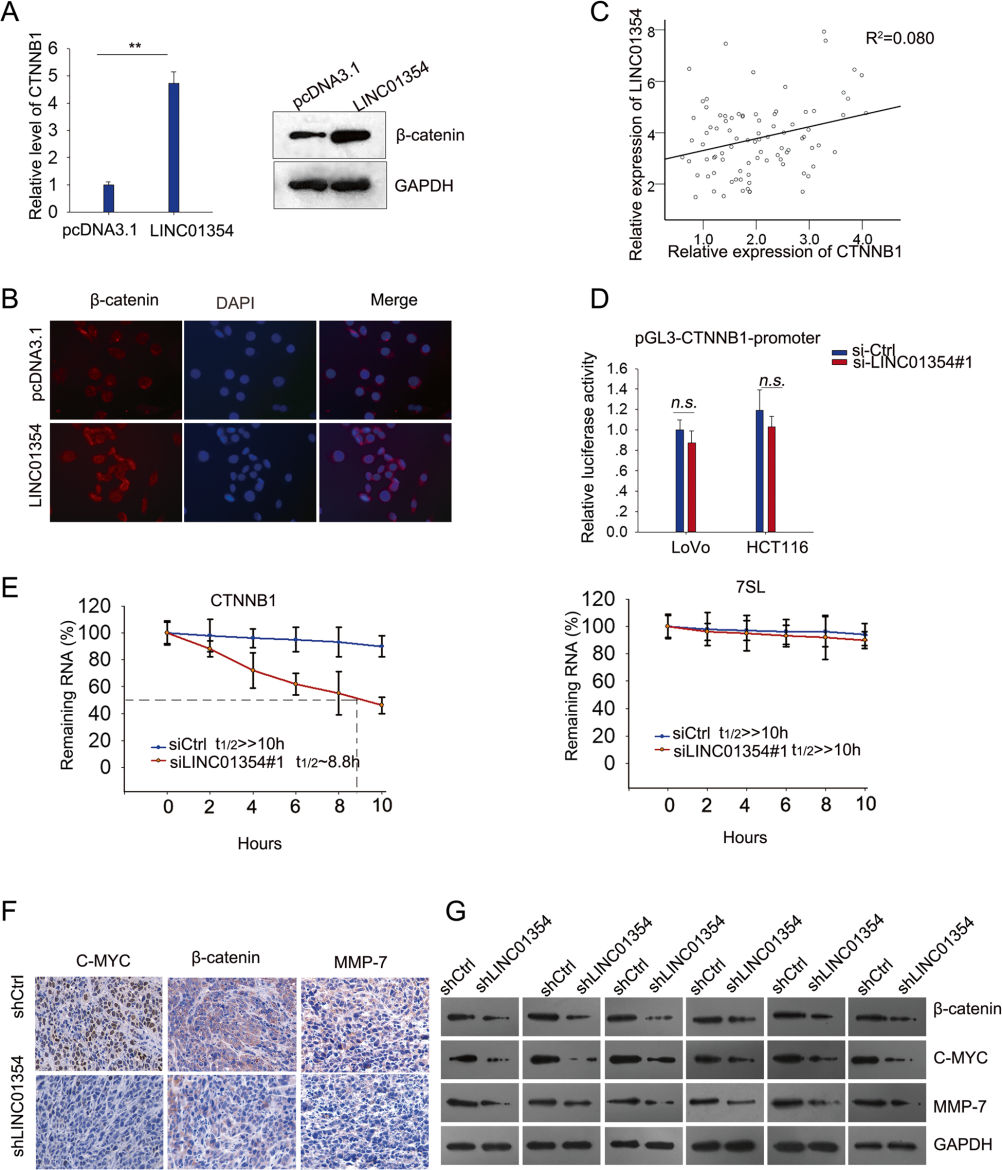
LINC01354 positively regulated β-catenin through modulating the CTNNB1 stability. **a** The mRNA level and protein level of β-catenin in HCT116 cells with overexpressing LINC01354 were measured by qRT-PCR and western blot. **b** The nucleus translocation induced by overexpression of LINC01354 was observed by IF assay. **c** A positive correlation between CTNNB1 and LINC01354 was tested by qRT-PCR. **d** Luciferase reporter assay demonstrated that the modulation of LINC01354 on CTNNB1 expression was not at transcriptional level. **e** Knockdown of LINC01354 decreased the stability of CTNNB1 with 7SL as negative control. **f**-**g** IHC and western blot results showed that silencing LINC01354 decreased the levels of c-Myc, β-catenin, and MMP-7 in vivo. Results were exhibited as the mean SD on the basis of three independent experiments, ^**^p < 0.01 and *R*^2^ = 0.080

We further tunneled how LINC01354 regulated Wnt/β-Catenin signaling. Seeing that β-catenin is the key protein in Wnt/β-Catenin pathway, we determined whether the expression of β-catenin was regulated by LINC01354. qRT-PCR and western blotting analysis illustrated that the mRNA level and protein level of β-catenin were significantly increased in response to the enforced LINC01354 level in HCT116 cells (Fig. 6a). Furthermore, it was found by IF assay that overexpressing LINC01354 could increase the expression of β-catenin in nucleus of HCT116 cells (Fig. 6b). By analyzing CRC tissues by qRT-PCR, we obtained a positive correlation between CTNNB1 and LINC01354 (Fig. 6c). To probe the regulatory mechanism of LINC01354 on β-catenin expression, we first evaluated the effect of LINC01354 on the promoter activity of CTNNB1 (the mRNA of β-catenin). As elucidated in Fig. 6d, no significant effect was observed, indicating that LINC01354 modulated the expression level of β-catenin at post-transcriptional level. It has been documented that lncRNAs could post-transcriptionally regulate gene expression through regulating mRNA stability. Thus, to probe the influence of LINC01354 on the mRNA stability of CTNNB1, we inhibited LINC01354 expression in LoVo cells, treated the cells with actinomycin D (or dimethylsulfoxide control) for 24 h to block new RNA synthesis, and then determined the loss of CTNNB1 over different periods. As presented in Fig. 6e, knockdown of LINC01354 shortened the half-life of CTNNB1. To examine whether LINC01354 could activate Wnt/β-catenin signaling pathway in vivo, we assessed the expression level of the key effectors in Wnt/β-catenin pathway in the xenografts by IHC and western blot analyses. It turned out that silencing LINC01354 could diminish the expression of c-Myc, β-catenin and MMP7 in vivo (Fig. 6f-g). These results suggested that LINC01354 regulated the level of β-catenin through modulating the stability of its mRNA CTNNB1.

### LINC01354 mediated the stabilization of CTNNB1 through interacting with RNA-binding protein hnRNP-D

**Fig. 7:**
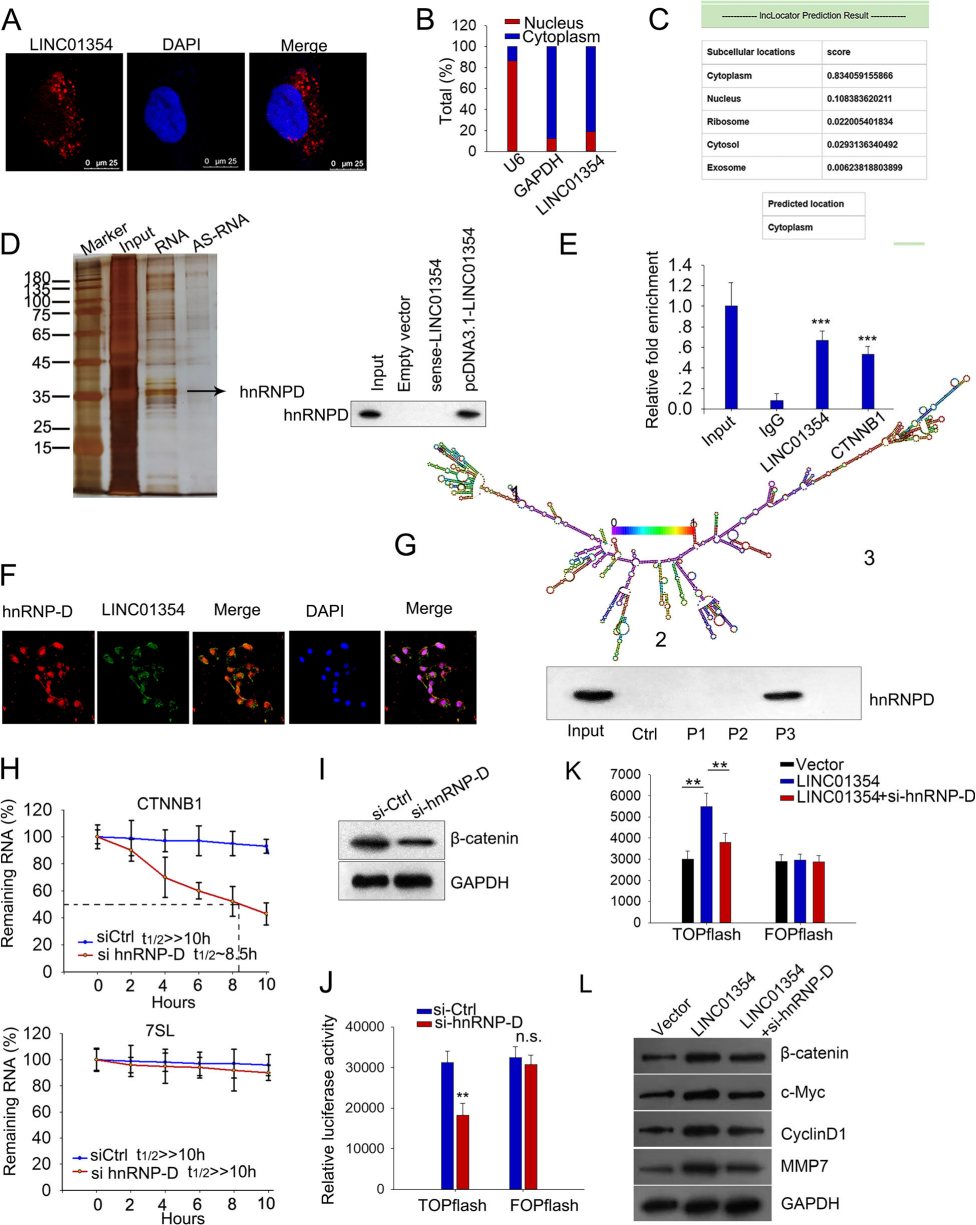
LINC01354 mediated the stabilization of CTNNB1 through interacting with RNA-binding protein hnRNP-D. **a** RNA FISH revealed the cytoplasm localization of LINC01354 in LoVo cells. **b**-**c** LINC01354 mainly presented in the cytoplasm by qRT-PCR analysis and such analysis was also verified on online. **d** hnRNP-D was identified among the potential binding partners with LINC01354 by RNA pull-down assays and mass spectrometry, and confirmed by western blotting. **e** RIP experiments in vitro exhibited a specific enrichment of LINC01354 and CTNNB1 with the hnRNP-D antibody but not IgG. **f** FISH assay demonstrated the overlapped location of LINC01354 and hnRNP-D expression in cytoplasm (**g**) The secondary structure of LINC01354 was presented and the fragment responsible for its interaction with hnRNP-D was identified by pulldown assay and western blot. **h** The stability of CTNNB1 could be decreased by silencing the level of hnRNP-D. **i**-**j** Knocking down hnRNP-D in LoVo cells, the level of β-catenin was significantly reduced as presented by western blot assay, and the Wnt/β-catenin was inactivated as depicted by TOP-Flash luciferase assay. **k** Silencing hnRNP-D abrogated the activation of overexpressing LINC01354 on Wnt/β-catenin as observed by TOP-Flash luciferase assay. **l** Western blot analysis showed that silencing hnRNP-D countervailed the inductive effect of overexpressing LINC01354 on the expression of β-catenin, c-Myc, CycinD1, and MMP7. Data were exhibited as the mean ± SD. All assays were carried out in triplicate. ^**^p < 0.01 and ^***^p < 0.001

To determine the precise mechanism by which LINC01354 regulated β-catenin expression so as to regulate Wnt/β-catenin signaling pathway in tumors, we examined the cellular location of LINC01354 because the activities and functions of lncRNAs depend on their subcellular distribution. Using RNA FISH (fluorescent in situ hybridization) technology, we determined the cytoplasm localization of LINC01354 in the LoVo cells (Fig. 7a). By isolating both nuclear and cytoplasmic RNA, we confirmed that LINC01354 was predominantly expressed in the cytoplasm by qRT-PCR (Fig. 7b), and same result was also obtained by analysis of online database (Fig. 7c). Based on these results, we drew the conclusion that LINC01354 was mainly located in cytoplasm. Since cytoplasmic lncRNAs are widely known to modulate gene transcription through interacting with RNA-binding proteins (RBPs) [23], we hypothesized that LINC01354 might regulate CTNNB1 through interacting with RBP. RNA pull-down assays were used to identify the LINC01354-interacting proteins. Several bands were identified by silver staining to be only pulled down by vitro-transcribed biotinylated LINC01354 sense transcript (Fig. 7d). Heterogeneous ribonucleoprotein D (hnRNP-D), was among the interacting proteins as analyzed by mass spectrometry, which was confirmed by western blotting (Fig. 7d). Next, we investigated the binding of hnRNP-D with LINC01354 and CTNNB1 in vitro by RNA-binding protein immunoprecipitation (RIP) experiments. Both LINC01354 and CTNNB1 were enriched in the precipitates of hnRNP-D antibody compared to a nonspecific antibody (IgG control) (Fig. 7e). IF and FISH assay presented the overlapped location of hnRNP-D and LINC01354 expression in cytoplasm (Fig. 7f). To figure out the specific binding domain on LINC01354 with hnRNP-D, we predicted the secondary structure of LINC01354 and carried out pulldown analysis. Results of western blot showed that segment P3 was responsible for the interaction of LINC01354 with hnRNP-D (Fig. 7g). Since hnRNP-D is a well-known RNA binding protein, mediating mRNA stabilization, we continued to investigate its effect on the mRNA stability of CTNNB1. We transfected HCT116 cells with si-hnRNP-D and then treated the cells with actinomycin D (or dimethylsulfoxide as control) over 24 h period to block new RNA synthesis. As shown in Fig. 7h, CTNNB1 mRNA stability could be reduced by silencing hnRNP-D. Furthermore, we silenced hnRNP-D in LoVo cells and disclosed the reduced level of β-catenin by western blot (Fig. 7i), and the attenuation of Wnt/β-catenin by TOP-Flash luciferase assay (Fig. 7j). Finally, we tried to examine whether LINC01354 could activate Wnt/β-catenin pathway through hnRNP-D. It was found that silencing hnRNP-D could rescue LINC01354 overexpression-activated Wnt/β-catenin (Fig. 7k). And western blotting analysis showed that the inductive effect of overexpressing LINC01354 on the downstream effectors involved in Wnt/β-catenin, including β-catenin, CCND1, c-Myc, and MMP7 could be countervailed by silencing hnRNP-D (Fig. 7l). These results suggested that LINC01354 could modulate the CTNNB1 stability through hnRNP-D so as to activate Wnt/β-catenin signaling.

## Discussion

Currently, lncRNAs have been clarified by emerging evidence as active biological molecules rather than ‘transcriptional noise’, which can drive carcinogenesis through regulating cellular processes, including promoting cell proliferation, preventing cell apoptosis, facilitating cell metastasis, modulating cell differentiation, and so on [24–27]. For instance, HULC, up-regulated by CREB, could contribute to the initiation and progression of liver cancer through interacting with miR-372 [28]; lncRNA PVT1 favors hepatocellular carcinoma cell proliferation and stem cell-like property by stabilizing NOP2 [29]. According to the location, in cytoplasm or nucleus, lncRNAs realize their function through the regulation of target genes at different levels, including transcriptional, post-transcriptional and epigenetic levels. In the nucleus, for example, lncRNA HOXA11-AS facilitates the proliferation and invasion of gastric cancer cells at epigenetic level through scaffolding the chromatin modification factors PRC2, LSD1, and DNMT1 [30]. In the cytoplasm, lncRNAs could exert their function through regulating protein localization, modulating mRNA translation or stability.

The key findings of the current study was that the overexpression and prognostic significance of LINC01354 in CRC tissues relative to corresponding adjacent non-tumor tissues from TCGA analysis and clinical specimens. Moreover, high level of LINC01354 expression is closely associated with tumor size, lymph node metastasis, TNM stage and distant metastasis. Combined with the results of gain- and loss-of-function assays, pathological and tumorigenic roles of LINC01354 were revealed, suggesting that LINC01354 promoted cell proliferation and migration/invasion and inhibited tumor cell apoptosis. Therefore, our investigations indicated that LINC01354 severed as an oncogene in CRC and can be explored as a potential prognostic and diagnostic indicator for CRC. For mechanism exploration, we revealed through the GO and KEGG analysis an association between LINC01354 and Wnt signal pathway and confirmed by TOP-Flash luciferase assay the activation of LINC01354 on Wnt/β-catenin pathway.

hnRNP-D, also called AUF1, was demonstrated to stabilize mRNAs that encode crucial regulators of cellular proliferation, differentiation and stress response. For instance, Wang et al. reported that AUF1 stabilized ZEB1 to induce epithelial-mesenchymal transition (EMT) so as to promote thyroid cancer [31]. AUF1 stabilized oncogene c-Myc to promote tumorigenesis [32]. In this study, we revealed that LINC01354 is mainly enriched in the cytoplasm, indicating its potential to regulate CTNNB1 expression through interacting with proteins. By performing RNA pull-down assay, we found that LINC01354 could interact with hnRNP-D, therefore modulated the mRNA stabilization of β-catenin mRNA. Furthermore, by analyzing the effect of LINC01354 on the degradation of β-catenin mRNA, we confirmed the role of LINC01354 in maintaining β-catenin mRNA stability. Besides, we found that LINC01354 exerted an oncogenic effect through hnRNP-D and that silenced hnRNP-D expression abrogated LINC01354’s function in CRC, inferring that LINC01354 functions in CRC in an hnRNP-D-dependent manner. What’s more, the activation of LINC01354 on Wnt/β-catenin signaling in CRC was obtained in vivo.

**Fig. 8:**
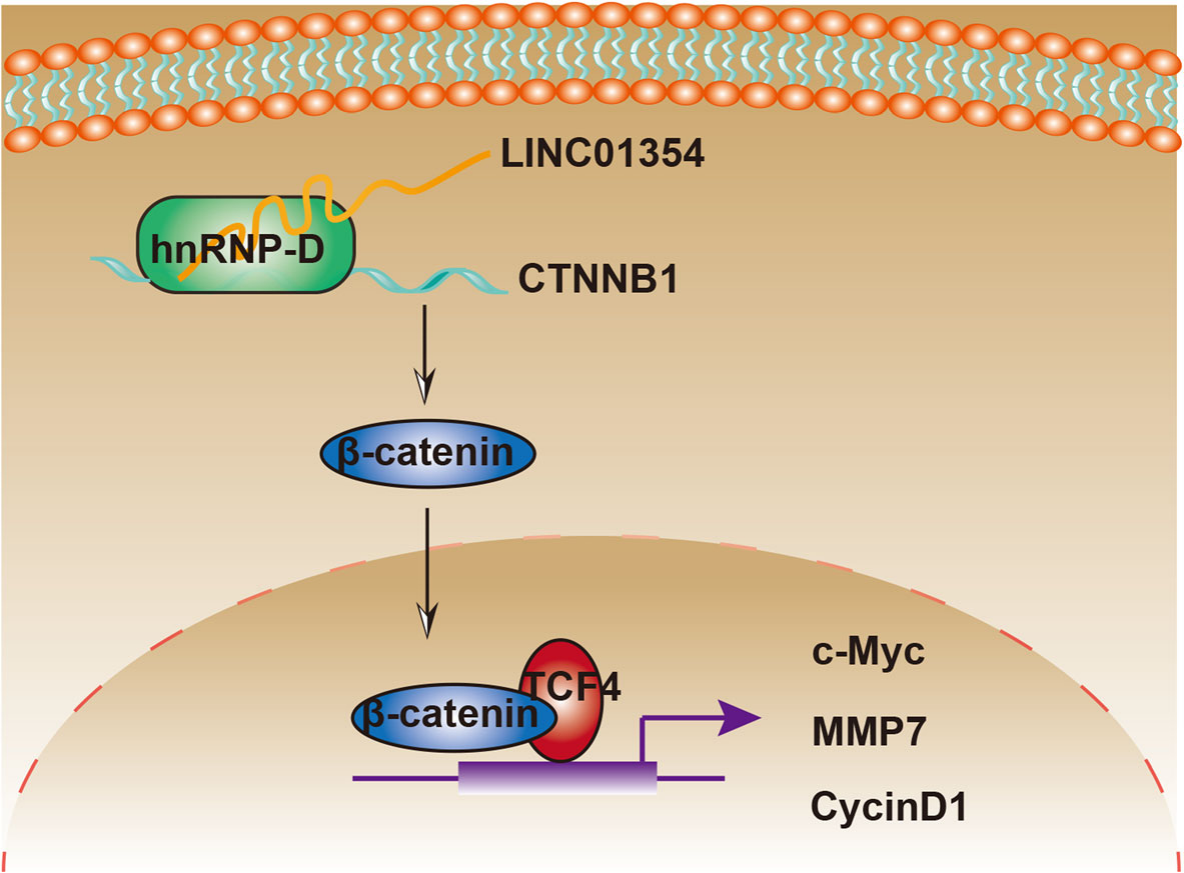
LINC01354 interacting with hnRNP-D contributes to the proliferation and metastasis via activating Wnt/β-catenin signaling pathway. Schematic representation of LINC01354 in colorectal cancer

In summary, our investigations depicted that LINC01354 was upregulated in CRC tissues and high level of LINC01354 was associated with poor prognosis of CRC patients. Gain- and loss-of-function assays illustrated that LINC01354 acted as an oncogene in CRC to facilitate tumor cell proliferation, inhibited apoptosis, and promoted cell migration and invasion in an hnRNP-D/Wnt/β-catenin-dependent manner (Fig. 8). These findings suggest that LINC01354 is a critical molecular target for tumor progression and is a potential diagnostic and prognostic biomarker for CRC.

## Conclusions

This study illustrated that LINC01354 was a novel prognostic marker in CRC. Functionally, LINC01354 facilitated CRC cell proliferation, migration and EMT. Mechanistically, LINC01354 stabilized CTNNB1 in an hnRNP-D-required way to activate Wnt/β-catenin pathway. Together, these findings provided innovative insights for the mechanism research and treatment progress for CRC.

## Additional file

Additional file 1: Controls were infected with empty vectors. qRT-PCR assays were performed to determine the transfection efficiency. (TIF 108 kb)

## Abbreviations

CRC: colorectal cancer
EdU: 5-ethynyl-2′-deoxyuridine
EMT: epithelial-mesenchymal transition
FISH: Fluorescence in situ hybridization
hnRNP-D: heterogeneous ribonucleoprotein D
IHC: Immunohistochemistry
LncRNAs: Long non-coding RNAs
RBPs: RNA-binding proteins
RIP: RNA immunoprecipitation
TCGA: The Cancer Genome Atlas
TNM: tumor-node-metastasis

## References

[1] Torre LA, Bray F, Siegel RL, Ferlay J, Lortet-Tieulent J, Jemal A (2015). Global cancer statistics, 2012. CA Cancer J Clin.

[2] Chaffer CL, Weinberg RA (2011). A perspective on cancer cell metastasis. Science..

[3] Guttman M, Amit I, Garber M, French C, Lin MF, Feldser D (2009). Chromatin signature reveals over a thousand highly conserved large non-coding RNAs in mammals. Nature..

[4] Nagano T, Fraser P (2011). No-nonsense functions for long noncoding RNAs. Cell..

[5] Fatica A, Bozzoni I (2013). Long non-coding RNAs: new players in cell differentiation and development. Nat Rev Genet.

[6] Flintoft L (2013). Structure and function for lncRNAs. Nat Rev Genet.

[7] Derrien T, Johnson R, Bussotti G, Tanzer A, Djebali S, Tilgner H (2012). The GENCODE v7 catalog of human long noncoding RNAs: analysis of their gene structure, evolution, and expression. Genome Res.

[8] Guttman M, Donaghey J, Carey BW, Garber M, Grenier JK, Munson G (2011). lincRNAs act in the circuitry controlling pluripotency and differentiation. Nature..

[9] Loewer S, Cabili MN, Guttman M, Loh YH, Thomas K, Park IH (2010). Large intergenic non-coding RNA-RoR modulates reprogramming of human induced pluripotent stem cells. Nat Genet.

[10] Schmitt AM, Chang HY (2016). Long noncoding RNAs in Cancer pathways. Cancer Cell.

[11] Huang JL, Cao SW, Ou QS, Yang B, Zheng SH, Tang J (2018). The long non-coding RNA PTTG3P promotes cell growth and metastasis via up-regulating PTTG1 and activating PI3K/AKT signaling in hepatocellular carcinoma. Mol Cancer.

[12] Li D, Chen Y, Mei H, Jiao W, Song H, Ye L, et al. Ets-1 promoter-associated noncoding RNA regulates the NONO/ERG/Ets-1 axis to drive gastric cancer progression. Oncogene. 2018. 10.1038/s41388-018-0302-4 PMC6117270 29773901

[13] Wang Y, Zeng X, Wang N, Zhao W, Zhang X, Teng S (2018). Long noncoding RNA DANCR, working as a competitive endogenous RNA, promotes ROCK1-mediated proliferation and metastasis via decoying of miR-335-5p and miR-1972 in osteosarcoma. Mol Cancer.

[14] Liu YW, Sun M, Xia R, Zhang EB, Liu XH, Zhang ZH (2015). LincHOTAIR epigenetically silences miR34a by binding to PRC2 to promote the epithelial-to-mesenchymal transition in human gastric cancer. Cell Death Dis.

[15] Gupta RA, Shah N, Wang KC, Kim J, Horlings HM, Wong DJ (2010). Long non-coding RNA HOTAIR reprograms chromatin state to promote cancer metastasis. Nature..

[16] Yoon JH, Abdelmohsen K, Kim J, Yang X, Martindale JL, Tominaga-Yamanaka K (2013). Scaffold function of long non-coding RNA HOTAIR in protein ubiquitination. Nat Commun.

[17] Wang X, Zhang L, Li M, Zhou X, Tan SK, Pastori C (2018). Serum long noncoding RNA HOTAIR as a novel diagnostic and prognostic biomarker in glioblastoma multiforme. Clinical cancer research : an official journal of the American Association for Cancer Research.

[18] Pasque V, Tchieu J, Karnik R, Uyeda M, Sadhu Dimashkie A, Case D (2014). X chromosome reactivation dynamics reveal stages of reprogramming to pluripotency. Cell..

[19] Han D, Wang M, Ma N, Xu Y, Jiang Y, Gao X (2015). Long noncoding RNAs: novel players in colorectal cancer. Cancer Lett.

[20] Tsai KW, Lo YH, Liu H, Yeh CY, Chen YZ, Hsu CW (2018). Linc00659, a long noncoding RNA, acts as novel oncogene in regulating cancer cell growth in colorectal cancer. Mol Cancer.

[21] Ning H, Albersen M, Lin G, Lue TF, Lin CS (2013). Effects of EdU labeling on mesenchymal stem cells. Cytotherapy..

[22] Raj A, van den Bogaard P, Rifkin SA, van Oudenaarden A, Tyagi S (2008). Imaging individual mRNA molecules using multiple singly labeled probes. Nat Methods.

[23] Lu Y, Liu X. The NF-kappaB-Responsive Long Noncoding RNA FIRRE Regulates Posttranscriptional Regulation of Inflammatory Gene Expression through Interacting with hnRNPU 2017;199(10):3571–82. 10.4049/jimmunol.1700091 PMC5672816 28993514

[24] Kung JT, Colognori D, Lee JT (2013). Long noncoding RNAs: past, present, and future. Genetics..

[25] Wapinski O, Chang HY (2011). Long noncoding RNAs and human disease. Trends Cell Biol.

[26] Deng G, Sui G (2013). Noncoding RNA in oncogenesis: a new era of identifying key players. Int J Mol Sci.

[27] Huarte M (2015). The emerging role of lncRNAs in cancer. Nat Med.

[28] Wang J, Liu X, Wu H, Ni P, Gu Z, Qiao Y (2010). CREB up-regulates long non-coding RNA, HULC expression through interaction with microRNA-372 in liver cancer. Nucleic Acids Res.

[29] Wang F, Yuan JH, Wang SB, Yang F, Yuan SX, Ye C (2014). Oncofetal long noncoding RNA PVT1 promotes proliferation and stem cell-like property of hepatocellular carcinoma cells by stabilizing NOP2. Hepatology (Baltimore, Md).

[30] Sun M, Nie F, Wang Y, Zhang Z, Hou J, He D (2016). LncRNA HOXA11-AS promotes proliferation and invasion of gastric Cancer by scaffolding the chromatin modification factors PRC2, LSD1, and DNMT1. Cancer research.

[31] Li S, Zhang H-Y, Du Z-X, Li C, An M-X, Zong Z-H (2016). Induction of epithelial-mesenchymal transition (EMT) by Beclin 1 knockdown via posttranscriptional upregulation of ZEB1 in thyroid cancer cells. Oncotarget..

[32] Huang J, Zhang A, Ho T-T, Zhang Z, Zhou N, Ding X (2016). Linc-RoR promotes c-Myc expression through hnRNP I and AUF1. Nucleic Acids Res.

