# Supplementary figures and images for: LINC01354 interacting with hnRNP-D contributes to the proliferation and metastasis in colorectal cancer through activating Wnt/β-catenin signaling pathway
Source: J Exp Clin Cancer Res. 2019 Apr 15;38:161. doi: 10.1186/s13046-019-1150-y (PMC6463672; doi:10.1186/s13046-019-1150-y)

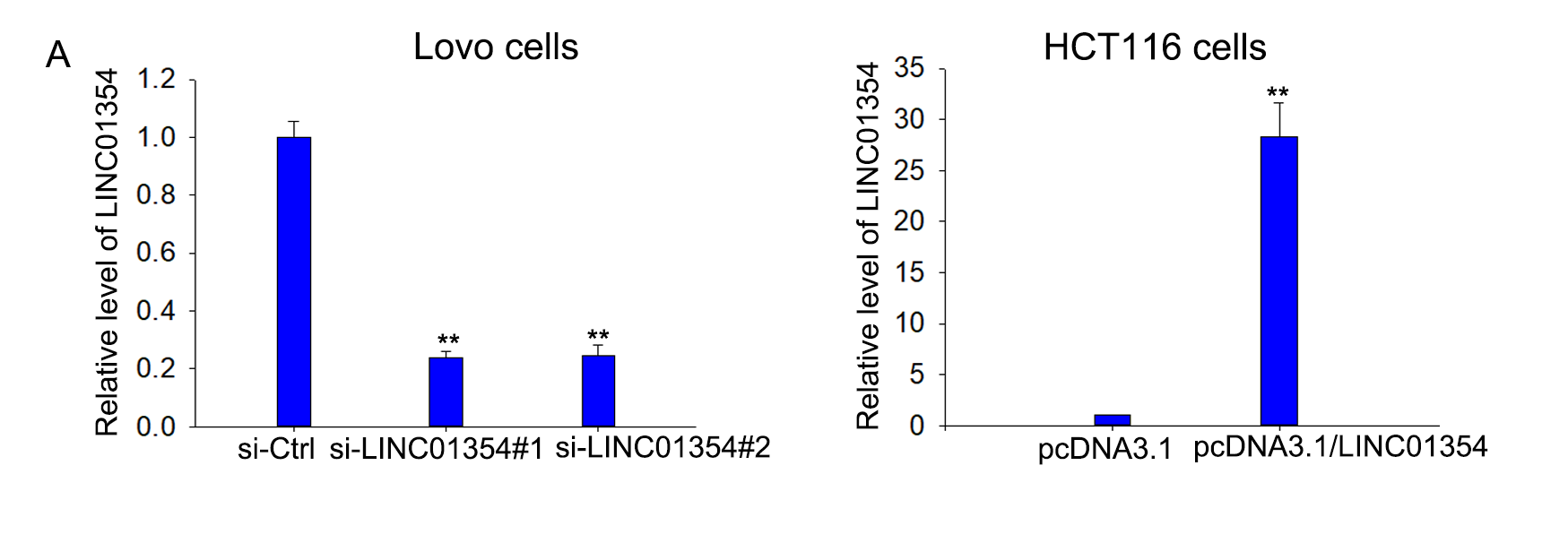

Supplement: Supplementary file 1 — Controls were infected with empty vectors. qRT-PCR assays were performed to determine the transfection efficiency. (TIF 108 kb) [file 13046_2019_1150_MOESM1_ESM.tif]
